# Highlight—The Deep Mystery of Mother’s Milk

**DOI:** 10.1093/gbe/evu227

**Published:** 2014-10-22

**Authors:** Danielle Venton

*“Figuring out how milk works is genuinely a massively important and challenging problem. We literally say milk is the Rosetta Stone of nourishment”*—Bruce German, UC Davis professor and food scientist.

One in eight babies is born premature, at least 3 weeks before his or her due date, each year in the United States. A large portion of infant deaths are attributed to early term births and surviving infants are more likely to suffer difficulty breathing, hearing loss, intellectual disabilities, and other health problems (Centers for Disease Control and Prevention, Office of the Associate Director for Communication, Digital Media Branch, Division of Public Affairs).

Monotremes on the other hand, representing the oldest lineage of mammals, begin feeding their young milk while they are still extremely immature, less than a month after conception. These tiny, practically embryonic, young thrive. Just how is a mystery, but the answer, researchers believe, is in the mother’s milk.

“By studying the milk of such species,” says Danielle Lemay, a bioinformatician and nutrition scientist at the University of California, Davis, “scientists can look for aspects unique to this premature-supportive milk to figure out how to [potentially] augment the nutrition of our own young when they are born too soon.”

A team of biologists from Australia recently investigated the protein components of the milk of extant monotremes to search for clues (Enjapoori et al. 2014). Their results, published in *Genome Biology Evolution*, highlight a unique protein, absent in modern mammals (i.e., marsupial and placental mammals), that selectively protects against certain bacterial species.

A long-standing point of discussion in the field, says Bruce German, milk researcher at UC Davis, is whether milk is primarily about providing nutrition to offspring or providing protection. The answer, he says, affects our view of breastfeeding, baby formula, and the nurturing of premature babies.

“The thing that really excites me is that this is so early on in evolution and yet at that point these tiny—somewhere between birds and mammals—they were already selecting for proteins that were antimicrobial,” German says.

Upon hatching from their egg, monotremes enter into a world teeming with microbiota. This they must navigate with essentially no immune system to speak of. Their sole source of nutrition and protection is their mother’s milk which comes from nipple-less ducts on her underside. It is a hairy, nonsterile environment in which the offspring feed by licking.

“It is quite possible to speculate the mother-to-hatchling transfer of pathogens,” write the authors. When tested in the lab, Monotreme Lactation Protein (MLP), a major component of monotreme milk, inhibited both opportunistic pathogenic *Staphylococcus aureus* and commensal *Enterococcus faecalis* bacteria. Upon other bacteria, such as *Escherichia coli*, *Pseudomonas aeruginosa*, *Staphylococcus epidermidis,* and *Salmonella enterica*, it showed no effect. Field reports show that platypus and echidnas are susceptible to common pathogenic Gram-positive and Gram-negative bacterial infections, such as *S. aureus* and *P. aeruginosa*. In fact, MLP may also be for the mother’s protection, as her mammary gland could be susceptible to a *S. aureus* infection, says primary author Ashwantha Kumar Enjapoori.

For the field work, Enjapoori et al. captured platypuses from river banks in Australia and Tasmania and echidnas from Tasmania, Australia, and New Guinea. The animals must be massaged to collect milk. Though the amount collected is not great (usually between 100 µl and 3 ml of milk depending on the animal and stage of lactation), the researchers collected enough from a few to have a small taste of the “liquid gold.”

The creamy white milk of the platypus is thick and intensely sweet, but pleasant, says Enjapoori. Echidna milk, which he did not get to taste, carries a lot of iron (likely because the hatchlings are so small their liver cannot store enough iron) and is pinkish in color.

Following collection, RNA was extracted from the milk cells for sequencing. Although MLP in platypus is closely related to the MLP in echidnas (and likely of the same origin), the researchers were somewhat surprised that they found no homologous protein in modern mammals. Given the unique presence of MLP in monotremes, Enjapoori believes that MLP is “necessary only for the monotreme way of primitive lactation.”

“As marsupial lineages evolved with nipples, and eutherians later adopted a longer gestation period supported by intrauterine development and placental support,” he says, “MLP was not needed.”

Nonetheless, the presence of such an effective and selective antimicrobial compound is exciting. “The beauty of it is you are not doing this for monotremes, however cute they are,” says German. “We want to know how to manipulate complex ecosystems in which bacteria are prominent players.”

Many antibiotics are broadly acting, often lethal to benign beneficial and commensal bacteria. A disrupted microbiome can lead to multiple problems, including digestion, weight fluctuations, and rheumatoid arthritis (Mayo Clinic, Center for Individualized Medicine).

“What you want is surgical precision,” German says, “something to support the elimination of bad bacteria, but not kill all the good guys. Since milk already has this strategy one could imagine that these components wouldn’t suffer from this problem of broad based antibiotics.”

Granted, a heavy dose of additional research is required before MLP could lead to new therapeutic approaches. Yet Enjapoori is hopeful. “Monotreme milk represents a rich source of untapped and potentially novel bioactive proteins with developmental or antimicrobial roles,” he says.

In the next stages of research, members of his team plan to analyze the genes and proteins expressed at different stages of monotreme lactation (the milk is known to change composition over time) to search for new bioactive compounds and new clues to the “Rosetta stone”-like mystery of milk.
